# The Saliva Proteome of Dogs: Variations Within and Between Breeds and Between Species

**DOI:** 10.1002/pmic.201700293

**Published:** 2018-02-23

**Authors:** Sabah Pasha, Taichi Inui, Iain Chapple, Stephen Harris, Lucy Holcombe, Melissa M. Grant

**Affiliations:** ^1^ Periodontal Research Group School of Dentistry Institute of Clinical Sciences College of Medical and Dental Sciences University of Birmingham and Birmingham Community Healthcare Foundation Trust Edgbaston Birmingham UK; ^2^ The WALTHAM Centre for Pet Nutrition Waltham‐on‐the‐Wolds Melton Mowbray Leicestershire UK

**Keywords:** breed, canine, dog, protein, saliva

## Abstract

Saliva is a complex multifunctional fluid that bathes the oral cavity to assist in soft and hard tissue maintenance, lubrication, buffering, defense against microbes, and initiating digestion of foods. It has been extensively characterized in humans but its protein composition in dogs remains poorly characterized, yet saliva composition could explain (patho) physiological differences between individuals, breeds and with humans. This pilot discovery study aimed to characterize canine saliva from two breeds, Labrador retrievers and Beagles, and to compare this with human saliva using quantitative mass spectrometry. The analysis demonstrated considerable inter‐individual variation and difference between breeds; however these were small in comparison to the differences between species. Functional mapping suggested roles of detected proteins similar to those found in human saliva with the exception of the initiation of digestion as salivary amylase was lacking or at very low abundance in canine saliva samples. Many potential anti‐microbial proteins were detected agreeing with the notion that the oral cavity is under continuous microbial challenge.

## 1 Introduction

Saliva is a complex multifunctional fluid released into the oral cavity from a variety of major and minor exocrine glands. Gingival crevicular fluid also flows into saliva contributing tissue and serum fluids to overall saliva composition. The major functions of human saliva have been described as: lubrication and physical protection, buffering; clearance of debris, maintenance of tooth integrity, antimicrobial activity, taste, and digestion.^1^ The proteinaceous components of saliva therefore have overlapping and multifunctional roles to fulfill these diverse functions. Given the breadth of physiological functions of saliva, it is likely that differences in composition between dog breeds and between dogs and humans may help explain physiological and patho‐physiological differences between them.

Early explorations of enzyme activities of dog saliva revealed a lack of salivary amylase in comparison to other mammals,^2^ that it contained high levels of non‐specific esterase, acid phosphatase, and pseudo‐cholinesterase activities.^3^ Immunoglobulin A was also reported to be the most abundant immunoglobulin.^4^ The techniques employed in these early papers were targeted approaches as the available technology at the time did not permit a global approach to assessing multiple components of canine saliva. Glycosylated proteins have also been reported to be common in canine saliva.^5^ Salivary glycoproteins have several roles including tissue lubrication and the aggregation of bacteria. The lubricating proteins comprise predominantly mucins, which are highly glycosylated and of high molecular mass in humans. Statherins, agglutinins, histidine‐rich proteins, and proline‐rich proteins are also known to aggregate bacteria in human saliva, facilitating their removal via deglutition and/or immune clearance.

In veterinary medicine, dog saliva has mostly been studied for cortisol determination,^6^ which varies with breed size, (where large dogs have lower salivary cortisol), between intact and castrated/neutered individuals,^7^ and with circadian rhythm.^8^ However, more recently, dog saliva has been used to measure C‐reactive protein^9^ and adiponectin^10^ for non‐invasive monitoring of systemic inflammation.

Comparisons with human saliva have highlighted that canine saliva has a higher pH (8.5 vs 6.5–7.5 in humans), buffering capacity, and mineral concentrations.^11^ These differences may contribute to dogs being less susceptible to dental caries but more susceptible to gingivitis due to higher calculus formation.^12^ Indeed, we have recently followed 52 dogs without an oral hygiene routine^13^, ^14^ over 60 weeks and 67% developed periodontitis at 12 or more teeth. Other groups have shown prevalence estimates for periodontal disease in dogs ranging between 44 and 100%.^15^, ^16^, ^17^, ^18^, ^19^

In depth analysis of the protein composition of canine saliva from three mixed breed individuals has been initiated by de Sousa‐Pereira et al. (2015).^20^ Using qualitative gel electrophoresis to compare to other mammals, including humans, they demonstrated that canine saliva contained a smaller proportion of lower molecular weight proteins. By using mass spectrometry based proteomics, ten common proteins were found across seven mammals: carbonic anhydrase, albumin, polymeric immunoglobulin receptor, prolactin‐inducible protein, lactoperoxidase, glutathione‐S‐transferase, and keratins 1, 9, and 10. De Sousa‐Pereira et al.,^20^ additionally reported that histatins and statherin were not found in dogs. In the present study, we set out to quantitatively analyze the protein composition of canine saliva for the first time in two differently sized breeds, Labrador retriever (large) and Beagles (medium), and to compare these with human saliva.

**Significance of the study**

This work illustrates for the first time, the variation within the protein composition of saliva for two different breeds of dog and a comparison between these two breeds and human saliva protein composition. Greater differences were seen between species, whereas there were high similarities between the breeds. There is inter‐individual variation, which may be of relevance when considering oral health in dogs as well as formulation of oral delivered medicines or foods.

## 2 Experimental Section

### 2.1 Dog Population

**Figure 1:**
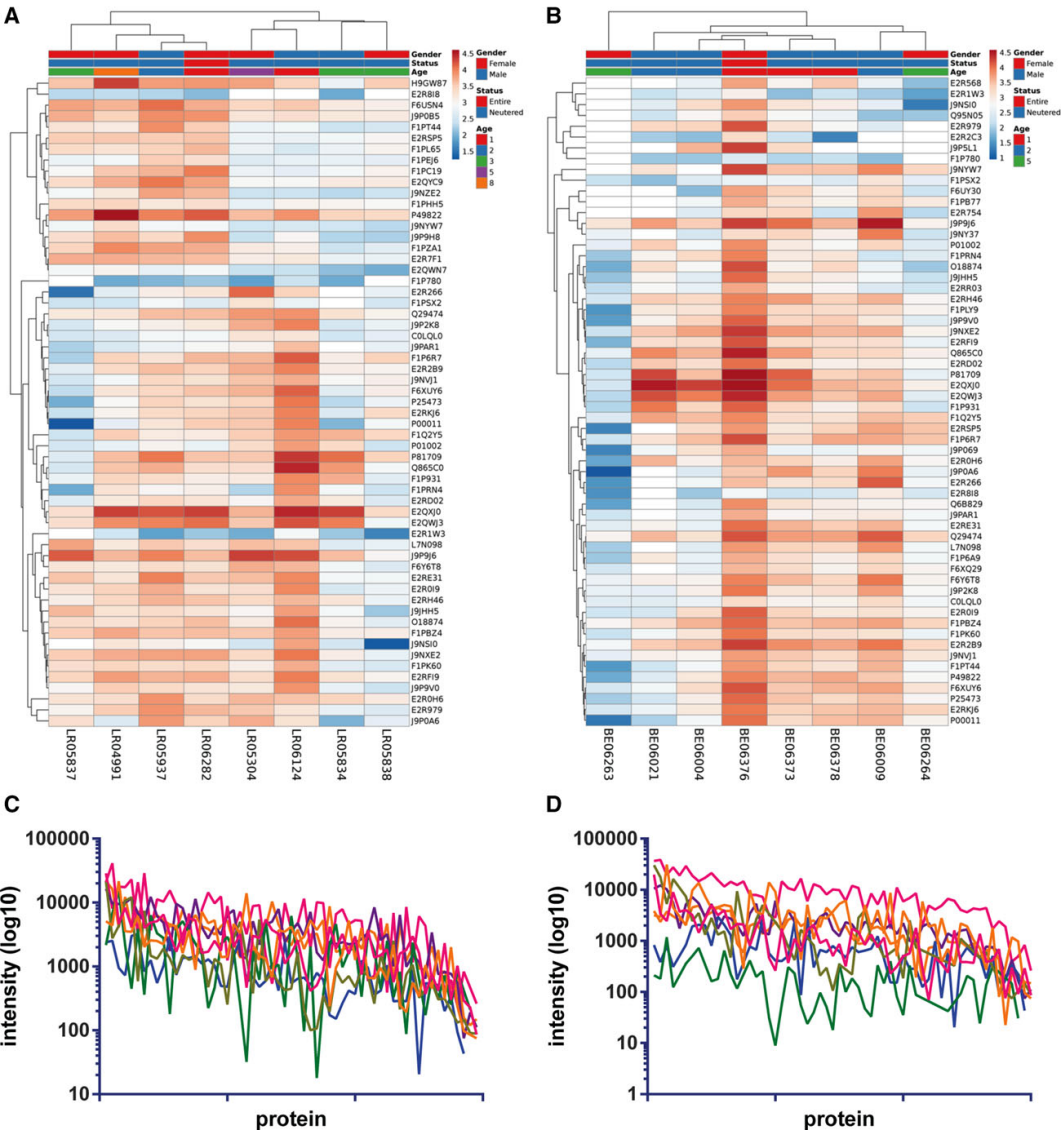
Heat maps of proteins identified in (A) Labrador retriever and (B) Beagle saliva, made with ClustVis, missing values shown in white. Protein intensities were log10 transformed and are displayed as colors ranging from red to blue as shown in the key. Both rows and columns are clustered using correlation distance and average linkage. Profile of proteins from (C) Labrador retriever (D) and Beagle; most abundant protein to right, colored lines represent individual dogs.

Sample collections described in this study were approved by The WALTHAM Centre for Pet Nutrition Animal Welfare and Ethical Review Body. ARRIVE guidelines for pre‐clinical studies were followed. The dogs were owned by WALTHAM and were housed at WALTHAM in kennels that exceeded the requirements of the Animal (Scientific Procedures) Act 1986 Code of Practice. Saliva samples were collected from 16 dogs of two breeds, on one day: eight Labrador retrievers and eight Beagles. The sample size was selected for pragmatic reasons and to allow for analysis via the isobaric tagging method described below. The animals were neutered, with the exception of one entire female per breed. The age ranges of the animals were from 1–5 years for Beagle and 1–8 years for Labrador retriever. The gender balance was five male: three female in the Beagle population and three male: five female in the Labrador retriever population. Individual ages and genders are shown in Figure 1A,B and Supporting Information, Figure S1. Animals received tooth brushing weekly and an oral health examination was carried out prior to the start of the trial to ensure all dogs had clinically healthy mouths with no signs of periodontal disease. All dogs received extensive training to ensure they were relaxed, responsive, and comfortable with the sample collection procedure. No drinking or eating took place an hour before sample collection. Dogs were excluded from the study if they had: 1) significant veterinary oral care, 2) systemic or oral antibiotic treatment, and 3) evidence of any extra‐oral bacterial infections.

### 2.2 Saliva Sampling

Saliva was collected onto SalivaBio Children's Swabs (as recommended by ref. 21 for recovery of protein sample) from eight Labrador retriever and eight Beagle dogs and eluted according to the manufacturer's instructions. The swab was used to sweep inside the mouth for 30 s to collect any pooled saliva. Sample collection took place approximately 8:00 am in the morning before the morning feed. Dogs had no access to water for at least for 10 min before the sample collection. The collected samples were placed on ice and immediately centrifuged at 12 000 × *g* for 10 min at 4 °C then stored at −80 °C until the analyses. All samples were confirmed to contain no evidence of any blood or food material.

### 2.3 Human Saliva Samples

Saliva samples were collected from five humans (age range 21–40 years, 60% female) chosen to be of a similar life stage to the dogs, on one day at approximately 9:30 am at the University of Birmingham School of Biosciences, by chewing on 3 g Parafilm M for 5 min and expectorating throughout the collection time into a 15 mL centrifuge tube. Samples were immediately centrifuged at 5000 × *g* for 5 min and stored at −80 °C until analysis. Participants self‐reported being in good health with no periodontal disease, use of anti‐inflammatories in the last week and had refrained from drinking or eating an hour before sample donation.

### 2.4 Sample Preparation

Protein content of saliva samples was estimated by the bicinchoninic acid assay.^22^ Approximately 100 μg saliva proteins were reduced with tris(2‐carboxyethyl)phosphine hydrochloride at 55 °C for 1 h and alkylated with iodoacetamide at room temperature, in the dark, for a further 30 min. Proteins were then digested overnight at 37 °C with Promega Gold Trypsin (1:40 tryspin:protein). Samples were cleaned prior to LC‐MS using ZipTips (5 μg max. binding capacity) according to the manufacturer's instructions.

For intra‐individual analysis across breeds resulting peptides were labeled with TMT10plex (Thermo‐Fisher Scientific) as per the manufacturer's instructions. Labeled peptides were combined and analyzed by LC‐MS/MS.

For comparison of human and dog saliva proteomes 100  μg of pooled samples from the three groups (human, Labrador retriever, or Beagle) were reduced and alkylated before digestion by trypsin (Trypsin Gold, Promega, UK). The samples were labeled with remaining TMT10plex unused labels from the breed experiments (Thermo‐Fisher Scientific) as per the manufacturer's instructions. Labeled peptides were combined and analyzed by LC‐MS/MS as technical duplicates.

### 2.5 Mass Spectrometry

Peptides were loaded on to a 150 mm Acclaim PepMap100 C18 column in formic acid (0.1% v/v). Peptides were separated over a linear gradient from 3.2 to 44% mobile phase B (acetonitrile with formic acid (0.1% v/v)) with a flow rate of 350 nL min^−1^. The column was then washed with 90% mobile phase B before re‐equilibrating at 3.2% mobile phase B. The column oven was heated to 35 °C. The LC system was coupled to an Advion TriVersa NanoMate (Advion) which infused the peptides directly into an LTQ‐Orbitrap Elite ETD (Thermo‐Fisher Scientific).

The mass spectrometer performed a full FT‐MS scan (*m/z* 380−1800) and subsequent CID MS/MS scans of the seven most abundant ions above an absolute signal intensity threshold of 5000 counts. Full scan mass spectra were recorded at a resolution of 60 000 at *m/z* 400 and ACG target of 1 × 10^6^ (maximum injection time 1 s). Precursor ions were fragmented in CID MS/MS with a normalized collision energy of 35% and an activation Q of 0.25. ACG target for CID MS/MS was 1 × 10^5^ (maximum injection time 50 ms). The width of the precursor isolation window was 2 *m/z* and only multiply charged precursor ions were selected for MS/MS. Spectra were acquired for 56 min.

A full FT‐MS scan (*m/z* 380–1800) was performed with subsequent HCD MS/MS scans of the seven most abundant ions that passed a minimum signal requirement of 5000 counts. The full FT‐MS scans were recorded at 120 000 resolution and ACG target of 1 × 10^6^ (maximum injection time 1 s). Precursor ions were fragmented in HCD MS/MS with a normalized collision energy of 38% and an activation time of 0.1 s. ACG target for HCD MS/MS was 1 × 10^5^ (maximum injection time 50 ms). The width of the precursor isolation window was 2 *m/z* and only multiply charged precursor ions were selected for MS/MS. FT first mass value was reduced to 120 *m/z* to account for TMT reporter ions.

The data were analysed using MaxQuant (v1.5.5.1). The UniProt *Canis lupus familiarus* database was used for dog proteins or the human database for human proteins. No microbial databases were included in the searches. The data were searched with the following settings: trypsin was selected as the enzyme with a maximum of two missed cleavages, 10 ppm mass accuracy for the precursor ion, fragment ion mass tolerance was set to 0.8 Da. Carbamidomethylation of cysteine and TMT addition to the *N*‐terminus and lysine residues were set as a fixed modification and deamidation of asparagine and glutamine and oxidation of methionine were added as variable modifications to the searches. Protein and PSM FDR were set at 1%. Proteins with more than two peptides per identification are included in further analysis.

### 2.6 Gel Electrophoresis

Individual saliva samples (10 μg protein) were mixed with an equal volume of Laemlli buffer (Sigma, UK) and heated to 92 °C for 5 min before loading on to a 12% gel (BioRad, UK) and separating at 150 V. Molecular weight was estimated by comparison to markers (Page Ruler, Thermo‐Fisher Scientific). Proteins were visualized by Instant blue stain (Expedeon).

### 2.7 Statistical Analysis

Data were analyzed with ClustVis^23^ to determine any clusters of individuals or species: to create heat maps, protein intensities were log10 transformed and both rows and columns are clustered using correlation distance and average linkage; NIPALS PCA is used to calculate principal components.

## 3 Results

**Figure 2:**
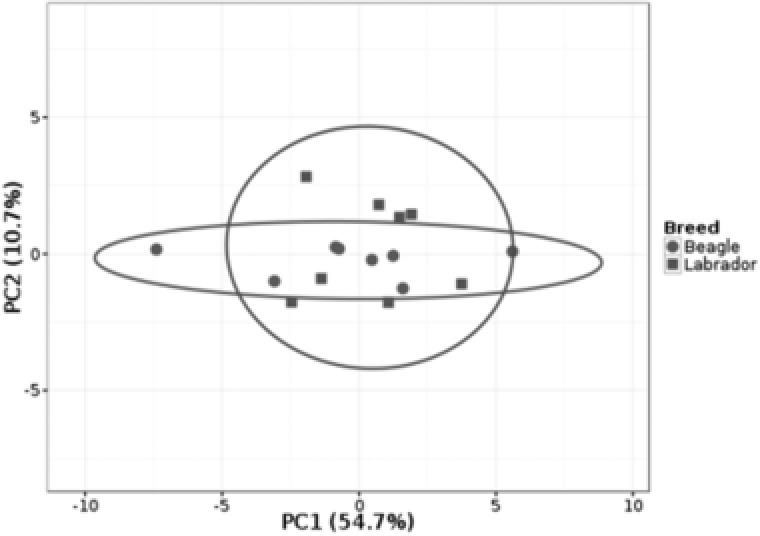
Principle component analysis of all dog saliva samples as analyzed for eight individuals per breed as in Figure 1. No scaling is applied to rows; NIPALS PCA is used to calculate principal components. X and Y axis show principal component 1 and principal component 2, respectively that explain 54.7 and 10.7% of the total variance, respectively. Prediction ellipses are such that with probability 0.95, a new observation from the same group will fall inside the ellipse. *N* = 16 data points.

**Figure 3:**
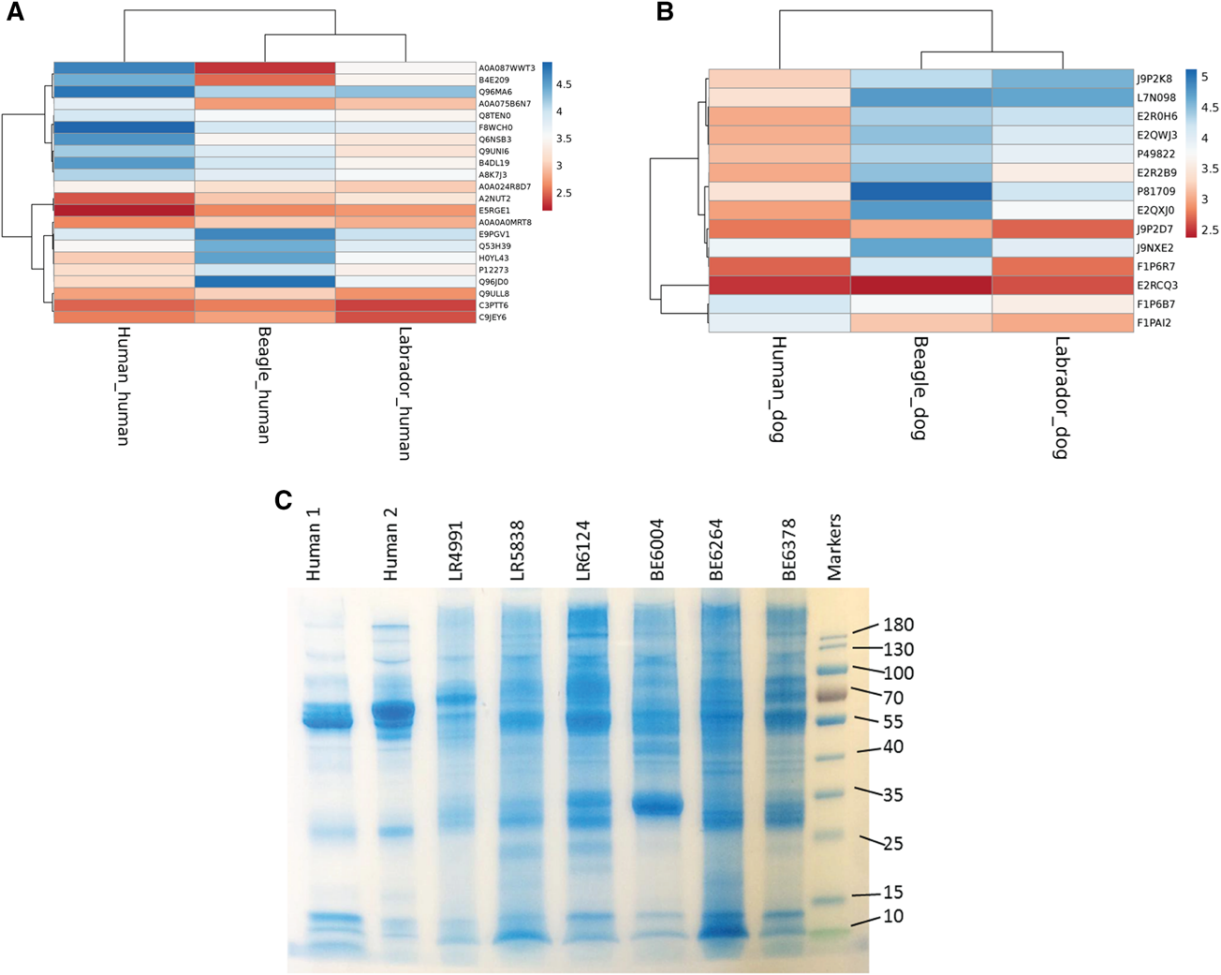
Heat maps of proteins identified in pooled human, pooled Labrador retriever and pooled Beagle saliva samples labeled with TMT tags. Data were searched against either (A) human and (B) canine databases. Heat maps were made with ClustVis, missing values are shown in white. Protein intensities were log10 transformed and are displayed as colors ranging from red to blue as shown in the key. Both rows and columns are clustered using correlation distance. C) Coomassie stained SDS‐PAGE gel of a subset of human and dog saliva samples.

Inter‐individual analysis of canine saliva by LC‐MS/MS analysis of the two different breeds, Labrador retriever and Beagle, revealed 59 and 60 salivary proteins, respectively. Supporting Information, Tables S1 and S2, list all of the proteins and Figure 1 visualizes the abundance of these in the individual samples. The heat maps in Figure 1A,B visualize all the proteins identified in the two breeds separately and are annotated for age gender and entire/neutered status per dog. The change in intensity across the different individuals is similarly displayed via a line graph showing that individual proteins behave differently between individuals (Figure 1C,D). These data demonstrate that within the samples analyzed, there is variation throughout the recorded profiles and this can also be seen for a more limited number of samples in Figure 3C and Supporting Information, Figure S1. No trends were observed in these small data sets pertaining to gender or age. By using principal component analysis to determine the contribution of the different proteins detected, BPI fold‐containing family A member 2 (BPIFA2) was found to be the main contributor to the first principal component (PC1) for both breeds. Similarly, when comparing the two different breeds there was a high degree of overlap with PCA (Figure 2).

To compare human to dog saliva, samples were combined into one multiplexed experiment and searched by using either the human or canine databases. This yielded 21 and 14 identifications, respectively (Figure 3). Notable differences in the detection were due to the lack of amylase in the dog database as compared to the human search (Figure 3A) and the abundance of mucin 7 in the dog samples (Figure 3B). Gel electrophoresis (Figure 3C) showed qualitative differences: in the human samples the putative amylase band at approximately 55 kDa dominates whereas in the canine samples there are many more bands detected potentially due to the lack of this dominating species.

## 4 Discussion

This study set out to explore the inter‐individual and inter‐breed proteomic variation of dog saliva and to compare the protein composition of dog and human saliva. Although only a small number of proteins have been discovered here these show a similar pattern to those found in three dog samples from mixed breed individuals investigated by de Sousa‐Pereira et al.^20^ Those authors report 244 proteins in dog saliva and 20 of these overlap with proteins with gene names discovered here. These proteins include carbonic anhydrase, albumin, polymeric immunoglobulin receptor, prolactin‐inducible protein, lactoperoxidase, and glutathione‐S‐transferase, which are the six of the ten proteins de Sousa‐Pereira et al.^20^ found across seven mammalian species. Notably keratins and alpha‐casein were not found amongst the proteins discovered in the present study; these were the remaining proteins found by de Sousa‐Pereira et al.^20^ in all seven mammalian species. The lack of keratins is possibly due to differences the duration of in saliva collection: here 30 s was used whereas de Sousa‐Pereira et al.^20^ used 4 min sublingually. Any movement over the longer time period may give greater opportunity for more epithelium to be incorporated in the sample. A larger number of proteins were detected in the study by de Sousa‐Pereira et al., than presented here. Modification of search terms to include phospho‐histidine, formed at the higher pH of dog saliva, and use of a mammalian database instead of a solely canine database did not improve peptide discovery (data not shown). Differences in the procedures used include use of in‐solution digest for this study and in‐gel digest for de Sousa‐Pereira et al.,^20^ which may have eliminated some interfering species. Further investigation into improvements in discovery of peptides and proteins in canine saliva are required. Additionally, further identifications could be made by including microbial proteins in the search; here only *Canis familiaris* proteins were used in the search as the dog oral microbiota genome database has not yet been published.^14^, ^24^

BPI fold‐containing family proteins (BPIFA2, BPIFB1, and BPL1) were found in apparent abundance in both Labrador retriever and Beagle saliva. These antimicrobial peptides, previously named palate, lung, and nasal epithelium carcinoma‐associated protein (PLUNC), are from the lipid transfer/lipopolysaccharide‐binding protein (LT/LBP) gene family and are involved in the recognition of bacterial products, activation of phagocytic cells, and olfaction.^25^

Proteomic studies of human saliva have demonstrated that there is considerable variation between individuals^26^ to a degree that outweighs variation in multiple donations from an individual. Additionally, Prodan et al.,^27^ when examining saliva from 268 healthy young humans and targeting particular enzyme activities (e.g., lysozyme) and protein levels (e.g., albumin), showed that there were small but significant differences between the genders.

Comparison of saliva from the two dog breeds showed that there was a greater similarity between dogs than with human saliva. Indeed, there seemed to be little impact by age and gender in this pilot study. The overlap of proteins identified was 63%. However, it appeared that there were more BPI fold‐containing proteins in the Beagle samples than in the Labrador retriever samples. Furthermore, the choice of sampling was different between the two species. A study by Golatowski et al. (2013)^28^ examining the difference between stimulated and unstimulated sampling techniques illustrated that there were differences in protein composition between these two types of samples; however the Pearson correlation was still 0.94. Thus the composition may be different due to the different choices of stimulation but it is likely that the changes will still be outweighed by change in species rather than change in stimulation; however this should be noted as a limitation of the study.

Mucins 5B, 7 and 19 were detected in both breeds of dog, however there was little detected in human saliva. There is conflicting evidence for its presence in human saliva: Rousseau et al. (2008)^29^ previously concluded that it was not present human saliva; whereas Zhu et al. (2011)^30^ could detect transcripts. MUC19 is a gel‐forming mucin and is implicated in preventing caries lesions as MUC19‐/‐ mice develop twice as main lesions in comparison to wild‐type mice.^31^ Dental caries is not common in dogs (prevalence 5.25% for one or more lesions^32^) and whilst diet many play a role, the underlying physiology of the species may also be of importance.

As reported previously, there appears to be no, or very little, salivary amylase present in canine saliva^3^, ^18^; however, isoamylase of pancreatic origin has been detected at a low level from unidentified breeds of dogs.^33^ Interestingly it has been suggested that amylase was acquired through the process of domestication: Freedman et al.^34^ reported a difference in the copy numbers of pancreatic amylase encoding genes separates between wolves and domesticated dogs. It is also noteworthy that multiple factors influence the salivary amylase level in human saliva, such as circadian rhythm, type of adsorbent used for collection, and mechanical stimulation in oral cavity.^35^ In comparison to human saliva it was only detected when searching against the human database. In general, the results from using the human or dog databases yielded different quantification. Highly conserved proteins appear to behave in similar manners: for example Actin and Annexin A1 have 100 and 92% homology, respectively and give identical quantification irrespective of the database used. However, the BPL1 shows 78% homology between the two species and returns different results when comparing the two databases: low quantities are detected in the human and Labrador retriever samples independent of the database used, however the Beagle results show higher levels within the dog database compared to the human.

**Figure 4:**
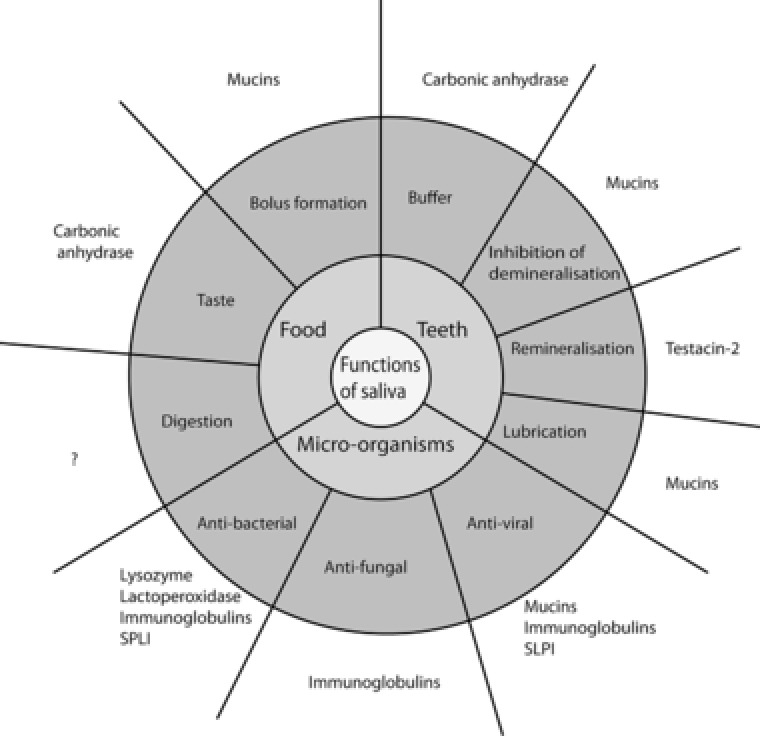
Schematic representation of canine saliva function based on ref. 30. Functions are derived from previous literature on human saliva for the proteins detected across Beagle and Labrador retriever samples. ? denotes no candidate proteins detected in this study.

Functional schemes of human saliva are common^36^ and so here the proteins discovered in canine saliva have been mapped on to a similar schema (Figure 4). In the present study, it is notable that there are no clear candidates for assisting in digestion or in remineralization. The presence of carboxylesterase and fatty acid‐binding protein imply degradative enzymes may be present in canine saliva. Presence of calcium‐binding proteins such as Protein S1008A and Testican‐2 suggests the same remineralization mechanisms exist between canine salivary proteins and those of humans, that is, proteins contribute to super‐saturate the minerals to maintain the equilibrium of hydroxyapatite to remain in enamel. A larger and deeper dataset may as yet reveal more candidates. Along with histatins and statherin, proline‐rich proteins were not detected, although there appear to be homologues in the *Canis lupus familiaris* genome (e.g., Uniprot entry J9P7N6_CANLF).

In summary, comparisons were made in this study between saliva samples collected from eight Labrador retriever dogs and eight Beagle dogs demonstrating inter‐individual variation within breeds and between breeds. Comparison against human saliva profiles confirms an earlier report demonstrating divergent profiles in these two species. The most apparent difference is the putative lack of digestive enzymes in canine saliva.

**Abbreviations**
BPI: bactericidal/permeability‐increasing
BPIF: BPI fold containing family
BPL: bactericidal/permeability‐increasing protein‐like
LT/LBP: lipid transfer/lipopolysaccharide‐binding protein
PC: principal component
PLUNC: palate, lung, and nasal epithelium clone
TMT: tandem mass tags

## Conflict of Interest

The authors have no conflict of interest.

## Supporting information

Supporting Information
