# Supplementary material for: The Saliva Proteome of Dogs: Variations Within and Between Breeds and Between Species
Source: Proteomics. 2018 Feb 23;18(3-4):1700293. doi: 10.1002/pmic.201700293 (PMC5969230; doi:10.1002/pmic.201700293)
Supplement: Supplementary file 1 — Supporting Information [file PMIC-18-na-s001.docx]

Supplemental Figure 1

Heatmaps of proteins identified in Labrador and Beagle saliva, made with ClustVis, with missing values shown in white. Protein intensities were log10 transformed and are displayed as colours ranging from red to blue as shown in the key. Both rows and columns are clustered using correlation distance and average linkage.


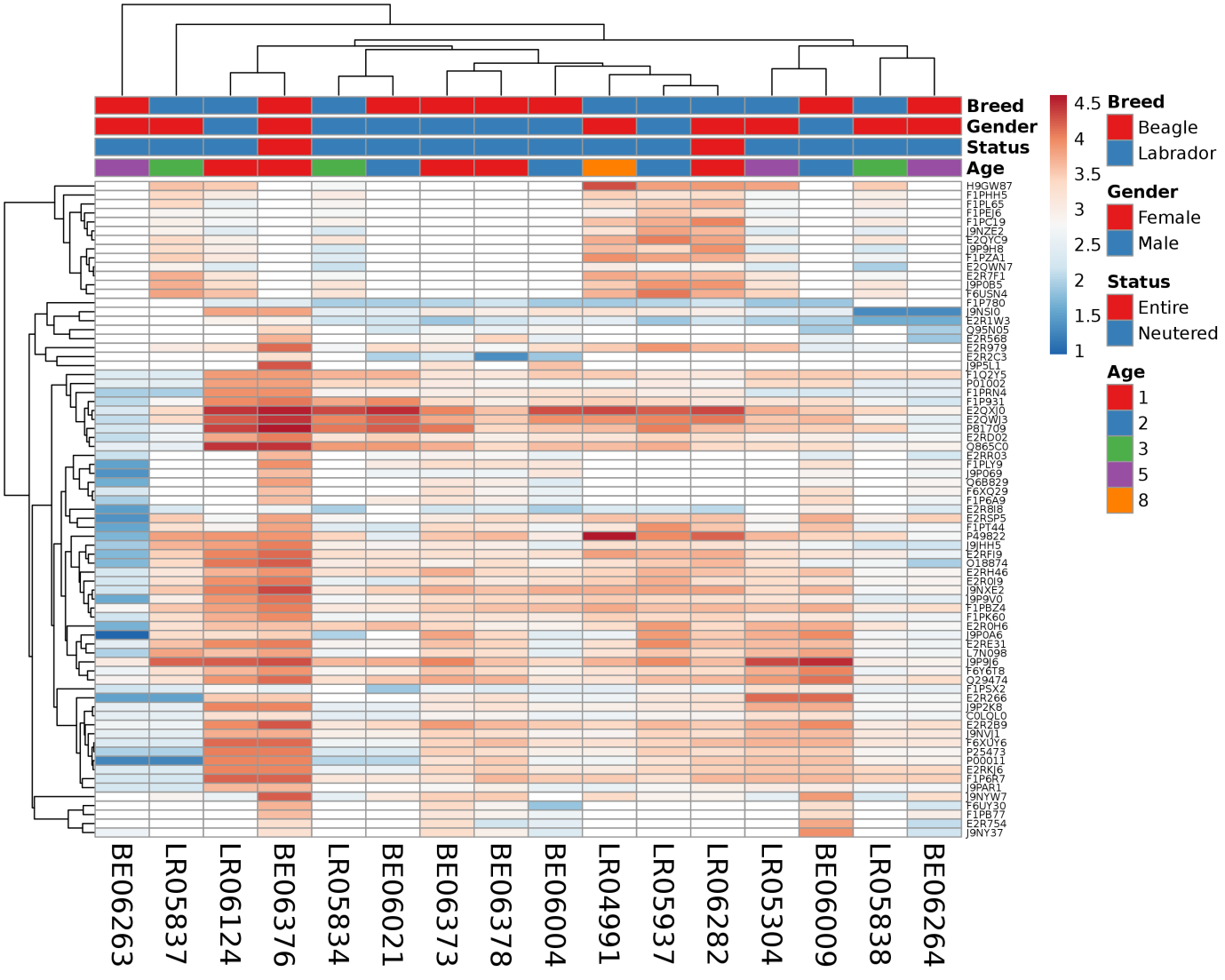


Supplemental Table 1. Labrador retriever data

| protein ID | Description | Gene name | LR04991 | LR05304 | LR05834 | LR05837 | LR05838 | LR05937 | LR06124 | LR06282 |
| --- | --- | --- | --- | --- | --- | --- | --- | --- | --- | --- |
| E2QXJ0 | BPI fold-containing family A member 2 | BPIFA2 | 21046 | 5102 | 21633 | 2160 | 2383 | 16380 | 28706 | 21614 |
| P49822 | Serum albumin | ALB | 41162 | 4368 | 2758 | 6796 | 2524 | 9678 | 8005 | 16255 |
| J9P9J6 | Ig heavy chain |  | 4613 | 21692 | 4332 | 15337 | 954 | 9541 | 17675 | 4096 |
| E2QWJ3 | BPI fold-containing family B member 1 | BPIFB1 | 8269 | 3549 | 10639 | 2170 | 727 | 9933 | 17388 | 12214 |
| P81709 | Lysozyme C, spleen isozyme |  | 4192 | 3479 | 12046 | 449 | 3003 | 11579 | 22933 | 3994 |
| H9GW87 | Transaldolase | LOC475937 | 20879 | 6148 | 588 | 3869 | 3224 | 6807 | 3049 | 7426 |
| Q865C0 | Carbonic anhydrase 6 | CA6 | 4016 | 2028 | 7423 | 361 | 835 | 5192 | 29018 | 2171 |
| F6USN4 | Ovostatin homolog 2 | LOC611455 | 4894 | 2564 | 1474 | 6350 | 1254 | 12302 | 3661 | 5391 |
| J9NXE2 | Beta-actin-like protein 2 | ACTBL2 | 5126 | 2496 | 1327 | 3309 | 553 | 6133 | 11224 | 6263 |
| E2RFI9 | Lactoperoxidase | LPO | 6463 | 2227 | 1893 | 2782 | 1018 | 4843 | 10353 | 5318 |
| F1P6R7 | Glutathione S-transferase Mu 4 | GSTM4 | 3177 | 4592 | 1331 | 180 | 2977 | 1740 | 15409 | 4484 |
| E2RE31 | Ig mu chain C region | IGHM | 1707 | 4202 | 768 | 3846 | 737 | 9597 | 9322 | 3665 |
| J9P0B5 | Lipocalin-1 | LCN1 | 3179 | 1613 | 1506 | 5116 | 1371 | 8197 | 2141 | 7683 |
| F1PBZ4 | NAD(P)H dehydrogenase [quinone] 1 | NQO1 | 5697 | 3717 | 1277 | 3438 | 1359 | 3760 | 6745 | 4255 |
| E2QYC9 | InaD-like protein | PATJ | 5267 | 895 | 1412 | 2289 | 1441 | 11452 | 889 | 6377 |
| Q29474 | Kallikrein |  | 1623 | 7215 | 1917 | 1553 | 1109 | 4108 | 7721 | 4254 |
| F6XUY6 | Fructose-bisphosphate aldolase | ALDOB | 1702 | 4764 | 473 | 223 | 1298 | 2734 | 14373 | 3887 |
| E2R2B9 | BPI fold-containing family B member 2 | BPIL1 | 2529 | 5271 | 1315 | 464 | 1033 | 4239 | 9721 | 3184 |
| O18874 | Minor allergen Can f 2 |  | 1774 | 1410 | 1076 | 2246 | 494 | 3132 | 12452 | 4247 |
| F1PZA1 | Isocitrate dehydrogenase [NADP] | IDH1 | 8808 | 1423 | 254 | 2890 | 480 | 6421 | 1247 | 5243 |
| E2R0H6 | Prolactin-inducible protein | PIP | 2027 | 4620 | 2817 | 1739 | 1433 | 7590 | 3800 | 2328 |
| F1Q2Y5 | Zymogen granule protein 16 homolog B | ZG16B | 3234 | 3270 | 4977 | 256 | 2639 | 1476 | 7242 | 1380 |
| E2R266 | Unconventional myosin-IXb | MYO9B | 465 | 13942 |  | 32 | 573 | 1334 | 3202 | 1682 |
| E2R0I9 | Glucose-6-phosphate 1-dehydrogenase | G6PD | 2999 | 2139 | 379 | 1751 | 682 | 5106 | 8844 | 1865 |
| F1P931 | angiopoetin | LOC607055 | 2808 | 1477 | 5441 | 617 | 479 | 2189 | 9080 | 1278 |
| E2R7F1 | Moesin | MSN | 5446 | 1313 | 593 | 5234 | 595 | 4511 | 1489 | 4136 |
| F1PC19 | Synaptonemal complex protein 2-like | SYCP2L | 3733 | 676 | 817 | 914 | 1138 | 5018 | 540 | 10329 |
| E2R979 | Spatacsin | SPG11 | 3238 | 3682 | 555 | 1069 | 420 | 7941 | 1607 | 4549 |
| E2RKJ6 | Leukocyte elastase inhibitor | SERPINB1 | 1110 | 3499 | 425 | 232 | 2397 | 2377 | 9826 | 2926 |
| L7N098 | Uncharacterized protein |  | 1585 | 4200 | 449 | 6202 | 544 | 3510 | 3842 | 2134 |
| E2RH46 | IgGFc-binding protein | FCGBP | 2561 | 2389 | 1674 | 1353 | 641 | 4757 | 4604 | 3768 |
| P25473 | Clusterin | CLU | 913 | 2927 | 210 | 96 | 666 | 2971 | 11519 | 1881 |
| J9P0A6 | Protein NDRG1 | DRG1 | 415 | 5277 | 100 | 2054 | 515 | 7169 | 1893 | 3145 |
| P00011 | Cytochrome c | CYCS | 507 | 3728 | 107 | 18 | 790 | 1647 | 10145 | 3240 |
| J9P2K8 | Mucin 7 | MUC7 | 866 | 5300 | 348 | 361 | 620 | 1242 | 9831 | 1564 |
| J9P9H8 | Hemoglobin subunit alpha | LOC100855558 | 4196 | 239 | 195 | 2147 | 173 | 2454 | 942 | 8753 |
| J9JHH5 | Immunoglobulin J chain | IGJ | 1901 | 956 | 850 | 4593 | 152 | 2255 | 6568 | 1692 |
| J9P9V0 | 14-3-3 protein epsilon | YWHAE | 3228 | 1555 | 555 | 975 | 264 | 2398 | 7840 | 1829 |
| F6Y6T8 | Polymeric immunoglobulin receptor | PIGR | 1571 | 5275 | 708 | 1698 | 420 | 2234 | 4576 | 1986 |
| F1PK60 | Protein-glutamine gamma-glutamyltransferase E | TGM3 | 2966 | 1266 | 474 | 2137 | 371 | 2300 | 5311 | 2716 |
| J9NVJ1 | Protein strawberry notch homolog 1 | SBNO1 | 751 | 4013 | 876 | 334 | 1233 | 2702 | 5191 | 1889 |
| E2RSP5 | F-box/WD repeat-containing protein 9 | FBXW9 | 3694 | 670 | 714 | 3247 | 948 | 3357 | 595 | 3760 |
| F1PT44 | Telomere-associated protein RIF1 | RIF1 | 1383 | 650 | 261 | 1737 | 362 | 8446 | 723 | 3318 |
| E2RD02 | Protein LEG1 homolog | C6orf58 | 1500 | 950 | 1561 | 440 | 420 | 2585 | 5693 | 1835 |
| F1PL65 | Sickle tail protein homolog | KIAA1217 | 1860 | 605 | 661 | 2513 | 1044 | 3207 | 390 | 4691 |
| P01002 | Double-headed protease inhibitor, submandibular gland |  | 569 | 2189 | 2513 | 330 | 286 | 1007 | 6897 | 608 |
| J9NZE2 | Armadillo repeat-containing protein 4 | LOC607539 | 1589 | 250 | 212 | 825 | 289 | 6432 | 271 | 4465 |
| F1PRN4 | Centrosomal protein of 135 kDa | CEP135 | 1809 | 192 | 743 | 88 | 286 | 987 | 8552 | 1224 |
| F1PHH5 | Mucin-5B | MUC5B | 2955 | 560 | 1064 | 2369 | 885 | 1485 | 1150 | 2039 |
| J9NSI0 | Arf-GAP with coiled-coil, ANK repeat and PH domain-containing protein 2 | ACAP2 | 1611 | 340 | 326 |  | 21 | 1182 | 6102 | 927 |
| J9PAR1 | Malate dehydrogenase | MDH2 | 371 | 1045 |  | 173 | 543 | 645 | 4325 | 892 |
| F1PEJ6 | Smoothened homolog | SMO | 748 | 451 | 559 | 665 | 541 | 3389 | 569 | 2068 |
| C0LQL0 | Protein S1008A | S100A8 | 419 | 1814 | 305 | 316 | 508 | 753 | 1727 | 763 |
| J9NYW7 | Uncharacterized protein |  | 2322 | 310 | 313 | 723 | 200 | 814 | 392 | 839 |
| F1PSX2 | Ig lambda-7 chain C region | IGLC7 | 299 | 2006 |  | 251 | 340 | 770 | 616 | 438 |
| E2QWN7 | Plastin-2 | LCP1 | 1028 | 238 | 133 | 834 | 94 | 558 | 243 | 806 |
| E2R1W3 | Transcription factor SOX-2 | SOX2 | 293 | 97 | 146 |  | 43 | 75 | 827 | 157 |
| E2R8I8 | Transient receptor potential cation channel subfamily V member 3 | TRPV3 | 228 |  | 91 | 211 |  | 190 |  | 120 |
| F1P780 | Exophilin-5 | EXPH5 | 86 | 75 | 89 |  |  | 112 | 263 | 149 |

Supplemental Table 2. Beagle data

| protein ID | Description | Gene name | BE06004 | BE06009 | BE06021 | BE06263 | BE06264 | BE06373 | BE06376 | BE06378 |
| --- | --- | --- | --- | --- | --- | --- | --- | --- | --- | --- |
| E2QXJ0 | BPI fold-containing family A member 2 | BPIFA2 | 19765 | 3177 | 30304 | 213 | 832 | 10499 | 36721 | 3863 |
| P81709 | Lysozyme C, spleen isozyme |  | 3400 | 2948 | 17644 | 183 | 386 | 12129 | 38584 | 2358 |
| J9P9J6 | Ig heavy chain |  | 2457 | 31107 | 5300 | 1188 | 792 | 10212 | 19659 | 3750 |
| E2QWJ3 | BPI fold-containing family B member 1 | BPIFB1 | 8961 | 4485 | 16203 | 124 | 311 | 5603 | 27520 | 3029 |
| J9P5L1 | Testican-2 | SPOCK2 | 3741 |  |  |  |  | 1747 | 19106 |  |
| Q865C0 | Carbonic anhydrase 6 | CA6 | 4016 | 2028 | 7423 | 361 | 835 | 5192 | 29018 | 2171 |
| E2R2B9 | BPI fold-containing family B member 2 | BPIL1 | 3206 | 9807 | 2464 | 506 | 1929 | 7193 | 18933 | 4517 |
| Q29474 | Kallikrein |  | 1454 | 13627 | 3511 | 723 | 2061 | 5454 | 14770 | 4886 |
| J9NYW7 | Uncharacterized protein |  |  | 7438 | 1333 |  | 2031 | 2913 | 17121 | 3129 |
| J9NXE2 | Beta-actin-like protein 2 | ACTBL2 | 5107 | 4316 | 2797 | 163 | 807 | 5276 | 21200 | 3599 |
| F1P6R7 | Glutathione S-transferase Mu 4 | GSTM4 | 3177 | 4592 | 1331 | 180 | 2977 | 1740 | 15409 | 4484 |
| F1PBZ4 | NAD(P)H dehydrogenase [quinone] 1 | NQO1 | 3992 | 5029 | 1530 | 643 | 2072 | 3026 | 11548 | 3823 |
| F6XUY6 | Fructose-bisphosphate aldolase | ALDOB | 1702 | 4764 | 473 | 223 | 1298 | 2734 | 14373 | 3887 |
| F1P931 | angiopoetin | LOC607055 | 2154 | 1894 | 9385 | 117 | 176 | 2049 | 12359 | 890 |
| E2R979 | Spatacsin | SPG11 | 2113 | 285 | 2033 |  |  | 1257 | 14674 | 596 |
| F6Y6T8 | Polymeric immunoglobulin receptor | PIGR | 806 | 9840 | 845 | 410 | 777 | 3409 | 7895 | 1987 |
| E2RH46 | IgGFc-binding protein | FCGBP | 1878 | 3745 | 2616 | 198 | 668 | 5342 | 7916 | 2258 |
| F1Q2Y5 | Zymogen granule protein 16 homolog B | ZG16B | 3234 | 3270 | 4977 | 256 | 2639 | 1476 | 7242 | 1380 |
| E2R266 | Unconventional myosin-IXb | MYO9B | 465 | 13942 |  | 32 | 573 | 1334 | 3202 | 1682 |
| J9P0A6 | Protein NDRG1 | DRG1 | 263 | 9149 |  | 9 | 410 | 6077 | 2873 | 2408 |
| E2RFI9 | Lactoperoxidase | LPO | 1730 | 1541 | 1580 | 57 | 405 | 1806 | 14652 | 1527 |
| E2RE31 | Ig mu chain C region | IGHM | 370 | 3978 | 766 | 306 | 508 | 3876 | 10720 | 2595 |
| E2RKJ6 | Leukocyte elastase inhibitor | SERPINB1 | 1110 | 3499 | 425 | 232 | 2397 | 2377 | 9826 | 2926 |
| O18874 | Minor allergen Can f 2 |  | 623 | 428 | 1655 | 54 | 87 | 803 | 16560 | 1939 |
| E2RSP5 | F-box/WD repeat-containing protein 9 | FBXW9 | 1633 | 5104 |  | 24 | 2892 | 1137 | 5961 | 2342 |
| J9P9V0 | 14-3-3 protein epsilon | YWHAE | 2069 | 1080 | 819 | 40 | 302 | 1985 | 12861 | 2497 |
| P25473 | Clusterin | CLU | 913 | 2927 | 210 | 96 | 666 | 2971 | 11519 | 1881 |
| P49822 | Serum albumin | ALB | 1059 | 2744 | 297 | 51 | 603 | 3492 | 8111 | 4427 |
| P00011 | Cytochrome c | CYCS | 507 | 3728 | 107 | 18 | 790 | 1647 | 10145 | 3240 |
| J9P2K8 | Mucin 7 | MUC7 | 866 | 5300 | 348 | 361 | 620 | 1242 | 9831 | 1564 |
| L7N098 | Uncharacterized protein |  | 319 | 6141 |  | 101 | 471 | 2319 | 6035 | 1974 |
| E2R0I9 | Glucose-6-phosphate 1-dehydrogenase | G6PD | 1726 | 1416 | 221 | 184 | 750 | 2109 | 11988 | 1143 |
| E2RD02 | Protein LEG1 homolog | C6orf58 | 1491 | 979 | 2825 | 124 | 602 | 1367 | 10870 | 882 |
| E2R0H6 | Prolactin-inducible protein | PIP | 837 | 5023 | 3948 | 47 | 647 | 2360 | 3606 | 907 |
| F1PLY9 | Fatty acid-binding protein, epidermal | FABP5 | 1326 | 1696 | 1063 | 33 | 703 | 2040 | 8439 | 1734 |
| J9NVJ1 | Protein strawberry notch homolog 1 | SBNO1 | 751 | 4013 | 876 | 334 | 1233 | 2702 | 5191 | 1889 |
| F1PK60 | Protein-glutamine gamma-glutamyltransferase E | TGM3 | 1158 | 1399 | 476 | 148 | 449 | 1678 | 9530 | 1580 |
| J9NY37 | Zinc transporter 3 | SLC30A3 | 229 | 8707 |  | 399 | 155 | 2073 | 1729 | 875 |
| J9JHH5 | Immunoglobulin J chain | IGJ | 313 | 503 | 725 | 82 | 152 | 1367 | 11016 | 953 |
| F1PT44 | Telomere-associated protein RIF1 | RIF1 | 504 | 3867 | 186 | 35 | 527 | 2237 | 5926 | 1299 |
| P01002 | Double-headed protease inhibitor, submandibular gland |  | 569 | 2189 | 2513 | 330 | 286 | 1007 | 6897 | 608 |
| F1PRN4 | Centrosomal protein of 135 kDa | CEP135 | 1809 | 192 | 743 | 88 | 286 | 987 | 8552 | 1224 |
| E2R754 | PDZ domain-containing protein 7 | PDZD7 | 295 | 5861 |  |  | 125 | 1914 | 1966 | 166 |
| F6UY30 | Dual specificity protein kinase CLK1 | CLK1 | 70 | 1904 |  |  | 179 | 2220 | 4893 | 728 |
| F1PB77 | Alpha-1,4 glucan phosphorylase | PYGL | 461 | 2080 |  |  | 982 | 1318 | 4117 | 783 |
| E2R568 | Keratin 4 | KRT4 | 365 | 388 |  |  | 76 | 716 | 4878 | 2768 |
| Q6B829 | Histone H4 |  | 241 | 695 |  | 42 | 661 | 1396 | 6662 | 959 |
| J9NSI0 | Arf-GAP with coiled-coil, ANK repeat and PH domain-containing protein 2 | ACAP2 | 1611 | 340 | 326 |  | 21 | 1182 | 6102 | 927 |
| F1P6A9 | Mucin 19 | MUC19 | 384 | 2181 | 1109 | 93 | 475 | 1598 | 4055 | 961 |
| F6XQ29 | Histone H3 | H3F3A | 384 | 1933 |  | 206 | 742 | 1679 | 3645 | 769 |
| J9PAR1 | Malate dehydrogenase | MDH2 | 371 | 1045 |  | 173 | 543 | 645 | 4325 | 892 |
| J9P069 | 14-3-3 protein zeta/delta | YWHAZ | 1375 | 723 | 468 | 24 | 489 | 452 | 4136 | 455 |
| E2RR03 | C-X-C chemokine receptor type 5 | CXCR5 | 386 | 273 | 600 | 137 | 185 | 773 | 4422 | 640 |
| C0LQL0 | Protein S1008A | S100A8 | 419 | 1814 | 305 | 316 | 508 | 753 | 1727 | 763 |
| Q95N05 | Carboxylesterase D1 | CESdD1 | 690 | 81 | 171 |  | 91 | 590 | 2438 | 424 |
| E2R2C3 | Glucose-6-phosphate isomerase | GPI | 75 |  | 101 |  |  | 176 | 1887 | 22 |
| F1PSX2 | Ig lambda-7 chain C region | IGLC7 | 299 | 1172 | 75 | 146 | 340 | 429 | 394 | 254 |
| E2R8I8 | Transient receptor potential cation channel subfamily V member 3 | TRPV3 | 92 | 978 |  | 31 | 223 | 165 | 499 | 224 |
| E2R1W3 | Transcription factor SOX-2 | SOX2 | 293 | 97 | 146 |  | 43 | 75 | 827 | 157 |
| F1P780 | Exophilin-5 | EXPH5 | 86 | 75 | 89 |  |  | 112 | 263 | 149 |
